# Clinical Series of Endogenous Klebsiella Endophthalmitis: Understanding an Aggressive Invasive Pathogen

**DOI:** 10.7759/cureus.110847

**Published:** 2026-06-14

**Authors:** Atiqah Wan Khairuzzaman, Jemaima Che Hamzah, Rona Asnida Nasaruddin, Mae-Lynn Bastion, Othmaliza Othman, Chenshen Lam, Niki Ho Wai Wye

**Affiliations:** 1 Department of Ophthalmology, Hospital Universiti Kebangsaan Malaysia, Kuala Lumpur, MYS; 2 Department of Ophthalmology, Faculty of Medicine, Universiti Kebangsaan Malaysia, Kuala Lumpur, MYS

**Keywords:** endogenous klebsiella endophthalmitis, invasive klebsiella pneumoniae syndrome (ikps), klebsiella liver abscess, klebsiella pneumoniae (kp), klebsiella pneumoniae liver abscess

## Abstract

We aim to delve into the characteristics of endogenous endophthalmitis caused by *Klebsiella* *pneumoniae*, focusing on the clinical manifestations, systemic risk factors, sources of the microorganism, and treatment outcomes among patients in our clinical series. All patients exhibited the classic symptoms of eye pain, redness, and reduced vision, with discernible risk factors of diabetes mellitus. In all cases, there were identifiable sources of bacteremia, most prominently liver abscess, followed by pneumonia and hand abscess. From the vitreous, positive cultures were obtained in all five cases, and all patients demonstrated bacteremia with positive *Klebsiella* growth. Late-stage unsalvageable presentations were observed in three cases, necessitating evisceration of the eye. One patient succumbed to sepsis with multiorgan failure. Despite prompt management of *Klebsiella* endophthalmitis, all patients in this clinical series had unfavorable visual or survival outcomes. Prevention in the form of good systemic control in patients with diabetes is crucial to prevent this life and sight-threatening complication.

## Introduction

Endogenous endophthalmitis is a vision-threatening ocular emergency typically resulting from hematogenous dissemination of systemic infection to the eye. It carries a particularly poor visual prognosis due to delayed diagnosis and the severity of the underlying systemic illness.

While endogenous endophthalmitis has been reported in young children, the majority of patients worldwide typically present in the fifth to sixth decades of life, with more cases among the male gender and a strong association with diabetes mellitus [1,2]. Although the causative organisms can vary from bacterial to fungal causes, literature indicates that Gram-positive cocci are the predominant pathogens in Western countries [1,2]. In contrast to the Southeast Asian region, Gram-negative bacteria are more commonly implicated, with *Klebsiella pneumoniae *being the most frequently isolated organism [3]. The pathophysiology commonly involves hematogenous spread from the infection site to the eye through the posterior segment vasculature. In some cases, direct spread of infection may occur in the setting of central nervous system infection via the optic nerve [4]. Prompt diagnosis and aggressive multidisciplinary management are essential to improve outcomes, although visual prognosis often remains guarded despite treatment.

We report five cases of endogenous endophthalmitis in which blood cultures isolated *K. pneumoniae*, confirming it as the causative agent of bacteremia. This study aims to report the clinical characteristics and outcomes of endogenous endophthalmitis in our center to contribute to the existing regional data and improve clinical awareness and management strategies.

This article was previously presented as a poster at the 39th Asia Pacific Academy of Ophthalmology (APAO) Congress on February 22, 2024.

## Case presentation

Case 1

A 52-year-old Chinese male with a background of diabetes mellitus presented with an acute onset of ocular pain and redness. His vision in the affected eye was markedly reduced to no light perception (NPL). Clinical findings included swollen and erythematous lids and conjunctival injection with chemosis. The anterior chamber demonstrated the presence of cells, fibrin, and hypopyon. As the fundus view was obscured, a B-scan was performed, revealing dense vitritis with loculations (Figure 1). Urgent intravitreal tapping was carried out, followed by intravitreal injection of ceftazidime and vancomycin. Vitreous culture subsequently yielded *Klebsiella *species, which was sensitive to ampicillin-sulbactam (Unasyn^TM^) and amoxycillin-clavulanate (Augmentin^TM^).

**Figure 1:**
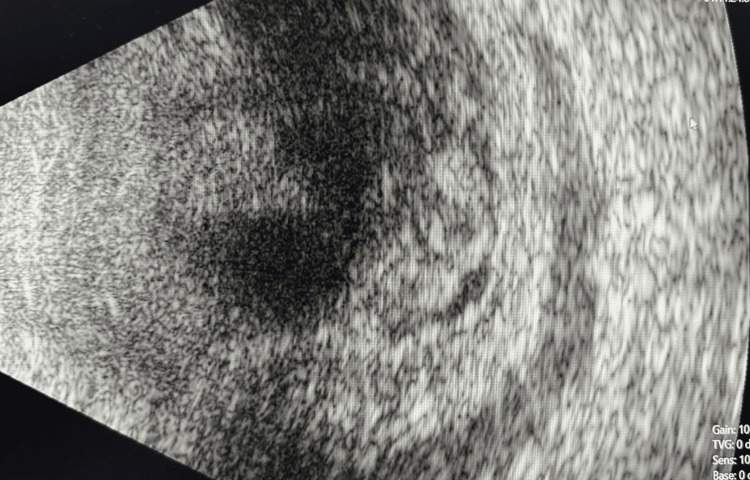
B-scan ultrasonography showing the presence of dense vitritis with loculations.

Upon further history, the ocular symptoms were preceded by a one-week history of fever, rigors, and chills, accompanied by abdominal discomfort. Systemic examination revealed pyrexia and a palpable, enlarged liver. Systemic evaluation for a possible source of infection included a contrast-enhanced computed tomography (CECT) scan of the abdomen, which showed evidence of multiple foci of liver abscesses, confirming endogenous endophthalmitis secondary to hepatobiliary seeding. The patient was co-managed with the gastroenterology team, who directed the management of the hepatic abscess. He subsequently underwent evisceration of the affected eye and was commenced on a course of intravenous antibiotics tailored to culture sensitivity.

Case 2

A 50-year-old Malay male with underlying diabetes mellitus and hypertension was referred for ophthalmic evaluation after developing acute ocular redness, pain, and discharge while being treated for *Klebsiella *bacteremia secondary to pneumonia. Ocular examination revealed that his vision was markedly impaired to hand movement (HM) with anterior segment findings of conjunctival injection, corneal edema, and anterior chamber inflammation with cells and hypopyon. B-scan ultrasonography demonstrated dense vitritis with loculated opacities. A diagnosis of endogenous endophthalmitis was confirmed; thus, an urgent vitreous tapping was performed, and intravitreal ceftazidime and vancomycin were administered.

Further systemic assessment revealed invasive *Klebsiella *syndrome with contrast-enhanced imaging identifying extensive multifocal abscesses in the liver, kidney, and pelvic region (Figure 2). The clinical course was complicated by septic encephalopathy and multiorgan failure. Despite prompt and intensive systemic and ocular management, he ultimately succumbed to complications of sepsis.

**Figure 2:**
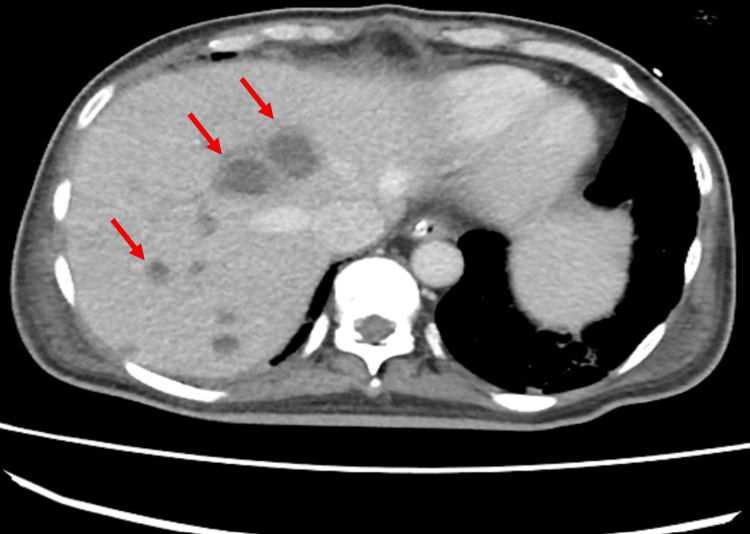
Contrast-enhanced computed tomography (CECT) scan revealing multiple hypodense lesions in the liver (arrowheads) suggestive of multifocal liver abscesses.

Case 3

A 66-year-old Malay male with underlying diabetes mellitus, hypertension, and end-stage renal failure (ESRF) presented with a two-day history of fever and shortness of breath. He was diagnosed with pneumonia and catheter-related bloodstream infection (CRBSI) by *Klebsiella *species, for which appropriate intravenous antibiotics were initiated. On day five of admission, he reported a new onset of left eye pain, progressive blurring of vision, and periocular swelling and redness. Ophthalmic evaluation revealed NPL vision in the affected eye, marked eyelid edema, conjunctival injection with chemosis, and the presence of hypopyon in the anterior chamber. B-scan ultrasonography demonstrated vitritis with loculations.

A diagnosis of endogenous endophthalmitis was established, and the patient underwent intravitreal tapping with administration of intravitreal antibiotics. Concurrently, he developed right hand abscess requiring surgical wound debridement. All cultures were obtained from the intraocular vitreous sample, central and peripheral blood stream, and the hand abscess yielded positive growth of *Klebsiella *species, indicating extensive hematogenous dissemination. Despite aggressive medical and intravitreal therapy, the patient's ocular condition continued to deteriorate with no signs of clinical improvement.

Due to the severity of the infection and lack of visual potential, evisceration of the left eye was performed. Intraoperatively, copious purulent material was noted within the globe (Figure 3), accompanied by areas of necrotic sclera. However, there was no evidence of overt globe perforation.

**Figure 3:**
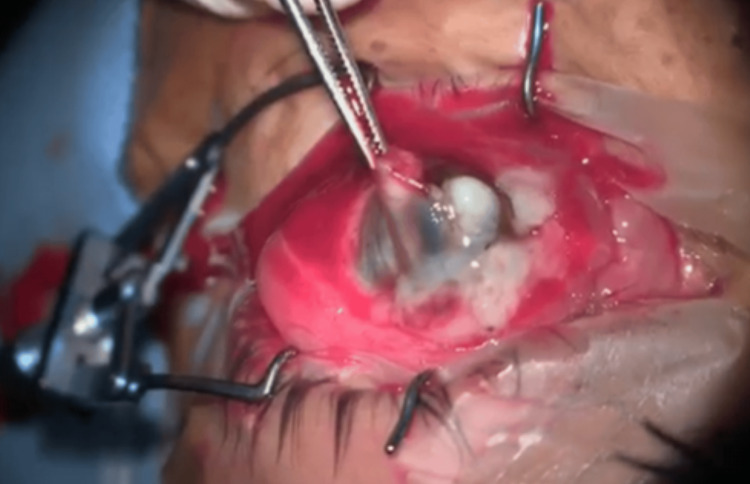
Intraoperative findings during evisceration, noted oozing of copious intraocular pus upon removal of the corneal button.

Case 4

A 51-year-old Malay woman with a background history of diabetes mellitus, hypertension, and dyslipidemia was undergoing treatment for a *K. pneumoniae *liver abscess when she was referred for ophthalmologic evaluation due to the acute onset of left eye pain, redness, and progressive visual loss over the preceding week. On examination, her visual acuity in the left eye was markedly limited to HM. There was diffuse conjunctival injection with the presence of fibrin covering the pupil, as well as hypopyon in the anterior chamber (Figure 4). Fundus view was obscured; hence, B-scan ultrasonography was performed, which revealed vitreous loculations. She was promptly treated with intravitreal antibiotics after samples from the vitreous were obtained as taken for culture and sensitivity. Due to the worsening of intraocular inflammation despite prompt medical therapy and poor visual prognosis, evisceration of the left eye was ultimately performed.

**Figure 4:**
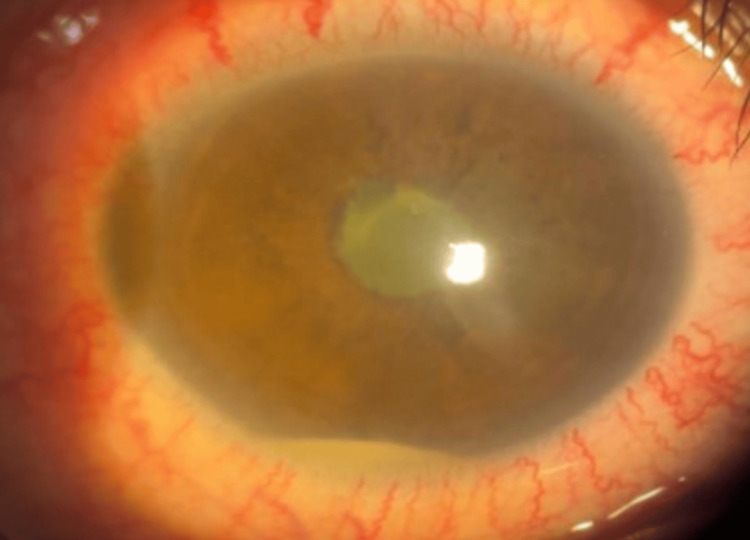
Anterior segment photograph demonstrating diffuse conjunctival injection, fibrin deposition obscuring the pupil, and hypopyon level indicating an intense anterior segment inflammation consistent with endophthalmitis.

Case 5

A 45-year-old Chinese male with underlying diabetes mellitus, hypertension, and dyslipidemia presented to the Emergency Department (ED) with high-grade fever and abdominal pain. He was diagnosed with biliary sepsis secondary to liver abscess (Figure 5), which necessitated ultrasound-guided percutaneous drainage. Culture of the abscess yielded *K. pneumoniae *species, and he was initiated on appropriate intravenous antibiotic therapy. Subsequently, he developed symptoms of left eye redness, pain, and progressively worsening vision. Ophthalmologic assessment revealed a reduced visual acuity of HM, conjunctival injection, and anterior chamber inflammation with hypopyon and fibrin obscuring the pupillary view; thus, fundus visualization was not possible. B-scan ultrasonography demonstrated dense vitritis with multiple loculations (Figure 5), consistent with endogenous endophthalmitis.

**Figure 5:**
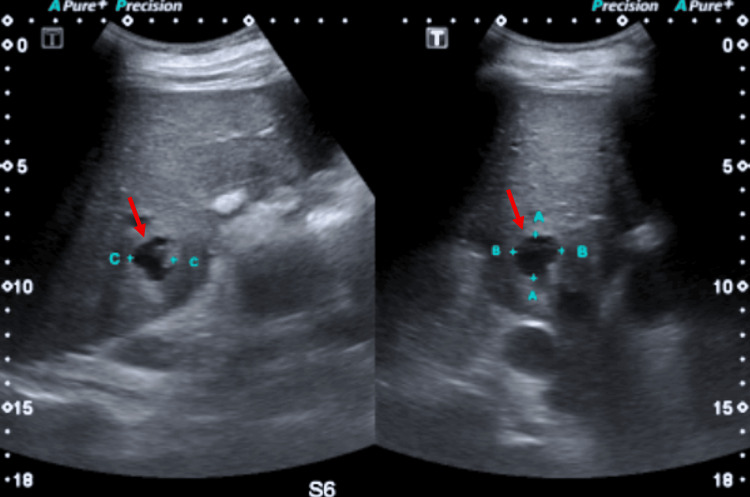
Ultrasound image demonstrating multiple well-defined hypoechoic lesions (arrows) within the liver, consistent with liver abscesses.

Given the presence of residual visual potential, the patient underwent an urgent trans pars plana vitrectomy (TPPV) with intravitreal antibiotics and silicone oil tamponade aimed at clearing the infectious vitreous load, delivering intravitreal therapy, and preserving the structural integrity of the globe. Intraoperative findings included dense vitreous pus with subretinal abscess along with areas of necrotic retina involving an extensive area of the macula, possibly reflecting the poor visual outcome in this patient despite the surgery. A summary of the salient clinical features of all five cases is presented in Table 1.

**Table 1:** Patient clinical characteristics; background history, clinical findings, treatment and outcomes. VA: Visual acuity, DM: Diabetes mellitus, NPL: No perception of light, HM: Hand movement, ESRF: End stage renal failure, CRBSI: Catheter-related bloodstream infection, TPPV: Trans pars plana vitrectomy

No.	Patient demographics	Comorbidities	Infective source(s)	Presenting visual acuity (VA)	Presenting ocular findings	Treatment and outcomes
Patient 1	51-year-old Chinese male	DM	Liver abscess	NPL	Conjunctival injection, chemosis, anterior chamber cells, fibrin, hypopyon, vitritis, and vitreous loculations	Intravitreal antibiotics, evisceration
Patient 2	50-year-old Malay male	DM, hypertension	Liver abscess	NPL	Periocular swelling, conjunctival injection, chemosis, anterior chamber cells, fibrin, hypopyon	Intravitreal antibiotics succumbed to sepsis
Patient 3	66-year-old Malay male	DM, hypertension, ESRF	Pneumonia, CRBSI, hand abscess	NPL	Periocular swelling, conjunctival injection, chemosis, hypopyon, vitritis, and vitreous loculations	Intravitreal antibiotics, evisceration
Patient 4	51-year-old Malay female	DM, hypertension, dyslipidemia	Liver abscess	HM	conjunctival injection, anterior chamber cells, fibrin, hypopyon, vitreous loculations	Intravitreal antibiotics, evisceration
Patient 5	45-year-old Chinese male	DM, hypertension, dyslipidemia	Liver abscess	HM	conjunctival injection, anterior chamber cells, fibrin, hypopyon, vitreous loculations	Intravitreal antibiotics, TPPV

## Discussion

The clinical presentation of endogenous endophthalmitis can vary widely, ranging from mild ocular discomfort, redness, and decreased vision to severe eye pain and profound visual loss, often reduced to light perception or even worse. The anterior segment may reveal conjunctival injection with chemosis, presence of cells, flare, fibrin, or hypopyon in the anterior chamber. Vitreous haze due to the presence of vitritis and possibly signs of chorioretinitis are also commonly documented in the posterior segment findings [5]. All five cases in our clinical series presented with some or all of these characteristic features at the time of diagnosis, with eye redness and pain being the predominant symptoms. In all five cases, vision at presentation was severely impaired, ranging from HM to NPL, reflecting the fulminant nature of the infection by *the Klebsiella *species.

Known risk factors for endogenous endophthalmitis include the presence of long-term indwelling catheters, intravenous drug use, chronic immunosuppression (such as in cancer, AIDS, or post-organ transplantation), debilitating conditions (such as diabetes mellitus, renal failure, and liver cirrhosis), and infection, such as endocarditis or urinary tract infections. In studies from Western countries, risk factors commonly include the use of indwelling urinary or intravenous catheters and systemic immunosuppression. In contrast, reports from Southeast Asia frequently associate the condition with uncontrolled diabetes mellitus and hepatobiliary disorders [5]. Consistent with existing literature, the majority of our patients had significant systemic comorbidities, primarily diabetes mellitus. Diabetes is a well-known risk factor for endogenous endophthalmitis due to immune dysregulation and disruption of the blood-retinal barrier, facilitating hematogenous spread to ocular tissues [6].

In addition, in our case series, all five patients developed endogenous endophthalmitis secondary to *Klebsiella *bacteremia, with liver abscess identified as the primary source in four out of five cases. These findings are consistent with previous reports identifying *Klebsiella*-related pyogenic liver abscess as a major risk factor for metastatic infections, including ocular involvement. A study by Hussain et al. in 2020 confirmed the fact that *K. pneumoniae-*related liver abscesses are significantly associated with metastatic dissemination to distant sites, leading to a range of focal septic complications, including meningitis and visceral abscess, and the most frequently reported metastatic manifestation is endogenous endophthalmitis [7].

Despite the timely detection of the disease and administration of systemic and intravitreal antibiotics, visual prognosis remained guarded for all of our patients. Of the patients reviewed in our case series, evisceration was required in three cases, all of whom presented with poor vision ranging from HM to NPL with severe intraocular inflammation and rapid deterioration of ocular infection despite treatment. Intraoperative findings in two of our cases revealed copious purulence, necrotic intraocular tissue, and scleral necrosis in one case, consistent with end-stage infection and poor globe salvageability. These findings underscore the importance of early detection and preserving ocular integrity.

Conversely, one patient underwent TPPV with silicone oil tamponade. This decision was made based on the presence of potential residual visual function, in which the surgery served not only to debulk the infectious material but also to facilitate the delivery of intravitreal antibiotics. While visual prognosis still remained guarded in this particular case due to extensive and severe intraocular infection spread, early surgical intervention can offer the best chance of anatomical and possible functional preservation in certain cases. Meanwhile, another patient in this case series, unfortunately, experienced a fatal outcome, succumbing to sepsis secondary to an aggressive form of *Klebsiella *infection, often referred to as invasive *Klebsiella *syndrome, which, in this case, has manifested as multisystem involvement, including severe pneumonia, intra-abdominal collection, and endophthalmitis. Despite early initiation of broad-spectrum antibiotics and maximal intensive care support, this patient developed sepsis and multiorgan failure, reflecting the rising threat of this hypervirulent strain [8].

## Conclusions

Despite prompt and aggressive management of *Klebsiella *endophthalmitis, all patients in this clinical series had unfavorable visual or survival outcomes. This underscores the fulminant nature of endogenous *K. pneumoniae *infections and their propensity for rapid intraocular and systemic deterioration despite timely multidisciplinary intervention. The findings in our clinical series highlight the importance of prevention as the most effective strategy in reducing morbidity, although limited by the relatively small sample size and single-center setting, which may restrict the generalizability of the findings.

Optimization of glycemic control in patients with diabetes mellitus is essential, given its strong association with *Klebsiella* bacteremia and metastatic infections. In addition, early recognition of at-risk patients and prompt multidisciplinary evaluation, including early ophthalmology consultation in high-risk patients with bacteremia, may facilitate earlier detection of ocular involvement and potentially improve outcomes. Endogenous *Klebsiella *endophthalmitis remains a devastating condition with significant visual and systemic consequences, reinforcing the need for heightened clinical vigilance and preventive strategies in high-risk populations.
